# The Impact of the COVID-19 Pandemic on Perinatal Loss Experienced by the Parental Couple: Protocol for a Mixed Methods Study

**DOI:** 10.2196/38866

**Published:** 2022-09-12

**Authors:** Loredana Cena, Alice Trainini, Nella Tralli, Luisa Silvia Nodari, Erika Iacona, Lucia Ronconi, Ines Testoni

**Affiliations:** 1 Observatory of Perinatal Clinical Psychology Department of Clinical and Experimental Science University of Brescia Brescia Italy; 2 Department of Philosophy, Sociology, Education and Applied Psychology University of Padova Padova Italy; 3 IT and Statistical Services, Multifunctional Centre of Psychology University of Padova Padova Italy

**Keywords:** COVID-19 pandemic, perinatal loss, bereavement care, mourning, anxiety, depression, posttraumatic stress disorder, COVID-19, pandemic, psychological, bereavement, miscarriage, stillbirth, neonatal death, parent, experience, coping, grief

## Abstract

**Background:**

At the beginning of 2020, mothers and fathers who experienced perinatal events (from conception to pregnancy and postpartum period) found themselves facing problems related to the emergency caused by the COVID-19 pandemic and the associated difficulties for health care centers in providing care. In the unexpected and negative event of perinatal loss (ie, miscarriage, stillbirth, and neonatal death) more complications occurred. Perinatal loss is a painful and traumatic life experience that causes grief and can cause affective disorders in the parental couple—the baby dies and the couple’s plans for a family are abruptly interrupted. During the COVID-19 pandemic, limited access to perinatal bereavement care, due to the lockdown measures imposed on medical health care centers and the social distancing rules to prevent contagion, was an additional risk factor for parental mental health, such as facing a prolonged and complicated grief.

**Objective:**

The main aims of this study are as follows: to investigate the impact of COVID-19 on mothers and fathers who experienced perinatal loss during the pandemic, comparing their perceptions; to evaluate their change over time between the first survey administration after bereavement and the second survey after 6 months; to examine the correlations between bereavement and anxiety, depression, couple satisfaction, spirituality, and sociodemographic variables; to investigate which psychosocial factors may negatively affect the mourning process; and to identify the potential predictors of the development of complicated grief.

**Methods:**

This longitudinal observational multicenter study is structured according to a mixed methods design, with a quantitative and qualitative section. It will include a sample of parents (mothers and fathers) who experienced perinatal loss during the COVID-19 pandemic from March 2020. There are two phases—a baseline and a follow-up after 6 months.

**Results:**

This protocol was approved by the Ethics Committee of Psychological Research, University of Padova, and by the Institutional Ethics Board of the Spedali Civili of Brescia, Italy. We expect to collect data from 34 or more couples, as determined by our sample size calculation.

**Conclusions:**

This study will contribute to the understanding of the psychological processes related to perinatal loss and bereavement care during the COVID-19 pandemic. It will provide information useful to prevent the risk of complicated grief and psychopathologies among bereaved parents and to promote perinatal mental health.

**International Registered Report Identifier (IRRID):**

DERR1-10.2196/38866

## Introduction

### Background and Rationale

The COVID-19 pandemic has seen almost 500 million people infected with the disease, including more than 6 million deaths globally [1]; it has also caused a global health, social, and economic crisis, with negative effects on the general population. Lockdown measures imposed to curb the spread of the infection [2] led to physical and emotional isolation, and this resulted in a global situation of uncertainty and psychological distress [3-5].

The COVID-19 pandemic also had collateral effects on mental health [6,7], with an increase in mental disorders in the general population [8] and mainly affecting the most vulnerable [9]. There are indeed indications that the adverse effects of health and social disasters are greater among vulnerable groups, such as the perinatal population [10]. The perinatal population is particularly vulnerable [11-13]—the period from conception to pregnancy and postpartum, which involves physiological, psychological, and social changes [14-16] and presents complex challenges for women [17] and men [18] who “transition to parenthood” [19]. Screenings over the prenatal and postnatal period [20] demonstrate that parents (mothers and fathers) may experience affective disorders [21] such as anxiety [22,23] and depression [24,25], also with comorbidity [26], with the risk of negative consequences for the mother’s health (eg, risks of miscarriage, pre-eclampsia, and gestational hypertension), and negative effects on the child’s development (premature birth; lower appearance, pulse, grimace, activity, and respiration scores; and low birth weight) [27] and on parents-infant relationships [28-30].

Due to the COVID-19 pandemic, these risk factors increased in the perinatal period; there were interruptions to the provision of perinatal health care services and changes to their structure [31,32]. During pregnancy, checkups and many routine outpatient visits were canceled [33,34], and there was a reduction in obstetric and psychological follow-ups. Delivery procedures during the pandemic also changed [35], with fathers not allowed to assist during labor. Lockdown and social isolation, together with restricted visits to maternity wards during hospitalization, in an effort to limit the risk of transmission of the virus, which reduced contact with family members, increased the negative psychological impact on the mother due to the lack of perceived social support [36,37]. Social support from the partner was also affected [38,39], as fathers were almost always not allowed to accompany mothers for checkups in health care centers. Preterm newborns were isolated from their mothers and fathers in the neonatal intensive care unit [40]. The pandemic restrictions also affected the immediate postpartum period, with interruptions in early dyadic relationships, particularly in mother-infant attachment [41]. International literature on COVID-19 effects reports mental health implications and distress in women during pregnancy [42], delivery, and postpartum [38,43], with an increasing prevalence of perinatal depression, anxiety [44-46], and posttraumatic stress disorders [47]. Pregnant mothers’ anxiety of attending checkups in clinics during routine prenatal care, due to the fear of being infected by SARS-CoV-2, and uncertainty about the effect of the virus on the fetus and infant, led to the postponing or cancelling of routine medical health care appointments [48], even though there was no consistent evidence of potential vertical intrauterine transmission of COVID-19 from mother to fetus [49,50]. The data collected by international researchers are controversial due to the lack of knowledge about the virus, which has generated many uncertainties about its long-term effects.

During the pandemic health emergency, maternal and fetal outcomes worsened globally, although there are limited data indicating that SARS-CoV-2 infection caused higher levels of adverse perinatal outcomes [51,52], measured in infected pregnant women compared to noninfected pregnant women [53]. Adverse outcomes include increased risks of perinatal loss [54,55]. Perinatal loss, that is, miscarriage (>20 weeks), stillbirth (>20 weeks gestation), or neonatal death (newborn in the first 28 days of life) [56], is an unexpected and complex negative life event, an experience that has always been poorly investigated. If we consider the period prior to the COVID-19 pandemic, more than 2 million perinatal deaths (*stillbirths*) and 2.9 million neonatal deaths occur worldwide every year [57]. However, The Lancet reports that not all of these deaths are recorded [58], and in the countries where the highest mortality occurs, the cause of these deaths is often not even identified. It should be noted that this high incidence has an economic impact on both global health and social systems [59]. Only recently did the World Health Organization [60] issue an operational guide to Maternal and Perinatal Death Surveillance and Response.

Perinatal death causes grief for the parental couple, requiring bereavement care [61]. In the international literature, “perinatal loss” refers to the death of the child in the perinatal period, but the term “loss” does not describe the parents’ state of mind and the complex psychological aspects of their suffering caused by this death. Perinatal loss is a painful and traumatic experience that can negatively affect a couple’s life; when the child dies, the plans for a new arrival in the family are abruptly halted, and the couple must process their mourning [62-65]. This interior processing of the grief over death is a necessary event, and the extent of their suffering depends on the affective investment of the parental couple in the child [66-69].

The possible negative consequences in terms of parental health can include affective disorders, such as anxiety, depressive, psychosomatic, and posttraumatic stress disorders [70]. Bereavement can lead to a crisis of faith [71], and the literature confirms that this can also occur in perinatal loss [72,73]. Perinatal loss is a biologically negative event, a particularly inexplicable experience; in the order of life events, children outlive their parents, hence the suffering of bereaved parents. Spirituality can serve as a coping mechanism to soften the complex painful feelings by helping mourners adapt to loss, and spiritual practices have been associated with better adjustment after the death of a child [71]. In a recent Italian study involving women who have experienced perinatal loss, it was found that religion helps them to accept grief and give meaning to such a tragedy [61].

There is a strong emotional impact also on the health care professionals working in maternity units [72,74]. It is important that these professionals understand parental perceptions to prevent the onset of psychopathologies, as perinatal mortality is an experience in which the early activation of the grief process is exacerbated by the circumstances surrounding this event [75].

During the pandemic period, mothers and fathers who suffered a perinatal loss found themselves experiencing further problems relating to COVID-19 [76], with the associated difficulties of the health system. Inpatient care for perinatal loss consists of bereavement care [77] according to specific clinical guidelines [78,79], which health care professionals were unable to follow during the pandemic to support parents. COVID-19 restrictions affected the provision of bereavement care compared to the period before the pandemic [80]. Personal protective equipment also prevented expressions of empathy from the operators, and support by means of physical contact was no longer permitted. There was also no time to train health workers adequately so that they could deal with the changes appropriately. These changes in care caused resentment among parents [81] and raised concerns over the possible negative impact on the long-term mourning process for parents and families [82] and increased risk of complicated grief [83].

The University of Padova and the University of Brescia propose a multicenter study, based in Italy, to optimize scientific knowledge in the field of studies of the effects of the COVID-19 pandemic on the perinatal period. There is a paucity of studies evaluating the psychological impact of the COVID-19 pandemic on couples experiencing the loss of their child in the perinatal period.

### Objectives

The main aims of this study are as follows:

- To investigate the impact of COVID-19 on mothers and fathers who experienced perinatal loss during the COVID-19 pandemic, comparing mothers’ and fathers’ perceptions
- To evaluate the change over time for fathers and mothers between the first survey after bereavement and the second survey, after 6 months
- To examine correlations of bereavement with anxiety, depression, couple satisfaction, spirituality, and sociodemographic variables. The main hypothesis is that the trauma was severe, to a greater extent for mothers, with outcomes of anxiety and depression. It is also hypothesized that more negative outcomes are related to difficult relationships, and this combination of traumatic experiences can lead to a crisis of faith, thus reducing the chances of resorting to religion as a coping mechanism
- To investigate which psychosocial factors negatively affect the mourning process, and identify the potential predictors of the development of complicated grief

## Methods

### Study Design

**Figure 1:**
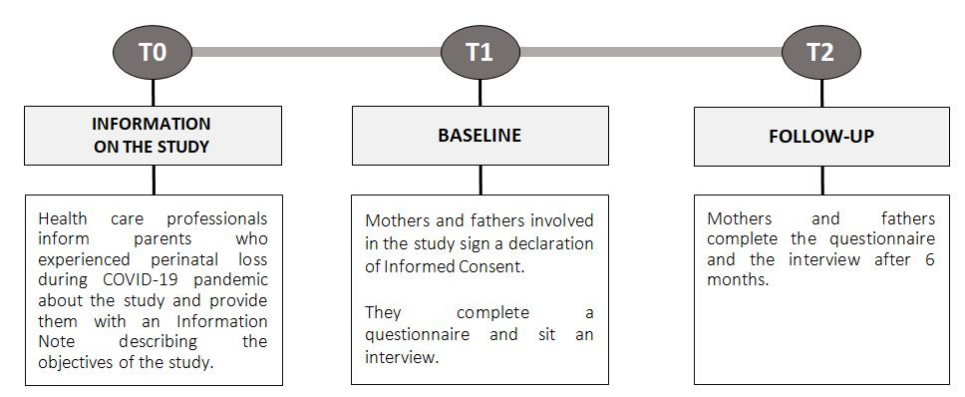
Timeline.

This longitudinal observational multicenter study is structured according to a mixed methods design, with a quantitative and qualitative part. The timeline of the whole procedure is shown in Figure 1. The study comprises two phases, which are a baseline (Figure 1: T1) and a 6-month follow-up (Figure 1: T2).

### Recruitment

Health care professionals (psychologists, psychotherapists, psychiatrists, midwives, etc) will conduct the study at health care centers throughout Italy (counseling centers, hospitals, etc) and the facilities involved and coordinated by the Observatory of Perinatal Clinical Psychology of the University of Brescia (Table 1). Among the mothers and fathers attending the health care centers, health care professionals will identify those who have experienced perinatal loss since the beginning of the COVID-19 pandemic (from March 2020) and will inform them about the study (Figure 1: T0). Health care professionals will provide mothers and fathers with an information note describing the aims of the study and will ask them to sign a declaration of informed consent if they intend to participate in the study. The first data collection time point (Figure 1: T1) will be after the death of the child, as soon as the parental couples are available to participate in the study, considering the difficulties due to the trauma for their perinatal loss. To protect their privacy, parents who agree to participate will be assigned a code with which they will become part of the study (participants can authorize the communication of their name to the research centers). Health care professionals will communicate the code or name of individuals participating to the Observatory of Perinatal Clinical Psychology of the University of Brescia. Both health care professionals and recruited participants will take part in this study voluntarily and without compensation.

**Table 1:** Health care professionals and health care centers, as well as facilities involved and coordinated by the Observatory of Perinatal Clinical Psychology of the University of Brescia.

Location	Unit type and name	Health care professionals
Bergamo	ASST^a^ Bergamo Est; Obstetrics OU^b^	1 PsyD^c^
Brescia	ASST Spedali Civili Hospital; Clinic and Family Centers	1 PsyD 2 Psychologists 2 Midwives
Como	Specialist Clinic of Perinatal Psychology	2 PsyDs
Enna	Umberto I Hospital; Obstetrics and Gynecology OUC^d^	1 PsyD
Florence	LHA^e^ of Toscana Centro; Family Clinic	2 PsyDs
Lodi	ASST Lodi; Obstetrics OU	1 Midwife
Mantua	ASST Mantua Carlo Poma Hospital; Maternal and Child Department Clinical Psychology; Obstetrics and Gynecology OUC and NICU^f^	2 PsyDs
Lecco	Arcobaleno and Pep Nursery School	1 Educationalist
Padua	Kairos Donna Association	1 Psychologist
Palermo	Buccheri La Ferla Hospital and Georgia Association	1 PsyD
Turin	LHA 3 of Turin; Specialist Centers of Perinatal Psychology	1 PsyD
Venice	Specialist Clinic of Perinatal Psychology	1 PsyD
Vicenza	ULSS8 Berica; Mental Health Department	1 Psychiatrist

^a^ASST: Azienda Socio Sanitaria Territoriale.

^b^OU: unit or department.

^c^PsyD: psychologist-psychotherapist.

^d^OUC: operating unit complex.

^e^LHA: local health authority.

^f^NICU: neonatal intensive care unit.

### Eligibility Criteria

Mothers and fathers who experienced perinatal loss during the COVID-19 pandemic form March 2020 and who are proficient in Italian are included in the study.

The main exclusion criterion is mental health—participants must not be diagnosed with a mental disorder by a psychiatrist and must not be undergoing psychiatric or psychopharmacological treatment.

### Materials and Procedure

In this Study, the participants (mothers and fathers) will be invited to participate in two phases of data collection. In the first phase (Figure 1: baseline—T1) they will perform the following:

- Complete a questionnaire (quantitative instrument) by the University of Brescia, which will be administered by the health professionals. The responses to the questionnaire will be entered by the health professionals directly into a web-based survey. Researchers at the University of Brescia will verify the quality of the data and coordinate the network of health professionals.
- Sit an interview (qualitative instrument) administered by specialized and trained researchers from the University of Padova, for consistency. The interview will be analyzed using thematic analysis [84].

In the second phase of data collection (Figure 1: follow-up—T2, after 6 months), mothers and fathers will be asked to complete the questionnaire and the interview by health care professionals. All data collected at each step will be deidentified and stored in a secure, password-protected drive with access only available to the research team members.

### Study Outcomes

The primary outcome for this study is the impact of the COVID-19 pandemic on the grief of mothers and fathers who experienced a perinatal loss during the pandemic. The secondary outcomes are the changes in social and couple relationships, maternal or paternal affectivity and satisfaction, spirituality, trauma, grieving strategies, and unhelpful or helpful factors. The tertiary outcomes relate to understanding the type of responsibility that parents ascribe to COVID-19 with respect to their perinatal loss.

#### Quantitative Measurement (Survey)

##### Sociodemographic Assessment Form for Mother or Father

The Sociodemographic Assessment Form has been designed to collect the mother’s or father’s sociodemographic data (ie, age, nationality, academic qualifications, professional status, economic situation, and current marital status) and anamnestic data (ie, date of birth; week of gestational age of the baby at the time of delivery; health facility where the birth took place; number of pregnancies; possible abortions; any mental disorders diagnosed; psychological therapies in progress; medication taken for depression, anxiety, or other problems; and perceived social support, eg, from family, friends, health services).

##### COVID-19—the Impact of Event Scale-Revised

The Impact of Event Scale-Revised [85] is a 22-item self-report tool that assesses subjective distress caused by traumatic events. Respondents are asked to identify a specific stressful life event and then indicate the degree of distress they felt over the following 7 days by each “difficulty” listed. Items are rated on a 5-point scale ranging from 0 (“not at all”) to 4 (“extremely”). The Impact of Event Scale-Revised yields a total score (ranging from 0 to 88), and subscale scores can be calculated for the Intrusion, Avoidance, and Hyperarousal subscales.

##### Prolonged Grief-13

The Prolonged Grief-13 [86] is a self-administered questionnaire consisting of 13 items, which evaluates the diagnosis of prolonged bereavement. The result is calculated based on an algorithm consisting of the following five criteria: (1) event of the loss; (2) separation distress (items 1-2); (3) duration (item 3); (4) cognitive, emotional, and behavioral symptoms (items 4-12); and (5) significant functional impairment, 6 months after loss (item 13). All five criteria must be met to diagnose prolonged grief disorder. The total score on the prolonged grief symptom scale is obtained by summing criteria (2) and (4).

##### Perinatal Assessment of Paternal or Maternal Affectivity

The Perinatal Assessment of Paternal or Maternal Affectivity [21] is a 10-item self-report questionnaire that investigates the following 8 dimensions: anxiety, depression, perceived stress, irritability or anger, relationship problems (eg, in couple, family, with friends, and at work), behavioral alterations of illness (eg, somatization, functional medical syndromes, and hypochondriac complaints), physiological disorders (eg, sleep, appetite, or sexual desire disorders), addictive disorders, and behavioral acting out. Some questions related to the paternity or maternity experience and the possible influence of sociocultural factors are included. The responses are indicated with an X on an analog line, with a rating from “Not at all” to “Very.” The line has small points, each of which corresponds to a score from 0 to 10. The tool allows us to identify fathers or mothers who have a significant risk of manifesting perinatal affective disorders. It is very simple to administer and quick to fill in, is suitable for different contexts, and is usable by professionals with different skills, both in public and private care settings.

##### Dyadic Adjustment Scale Brief Version

The Dyadic Adjustment Scale brief version [87] is a shortened version of the Dyadic Adjustment Scale. It is a self-report tool for evaluating couple satisfaction and is composed of the following 4 items: three items are on a 6-point Likert scale, ranging from 0 (all the time) to 5 (never), while the final item is on a 7-point scale ranging from 0 (extremely unhappy) to 6 (perfect).

##### Daily Spiritual Experiences Scale

The Daily Spiritual Experiences Scale [88] is a self-report tool composed of 16 items with 6-point Likert response (1=never; 6=many times), and it examines the dimension of the perception of the transcendent in the individual and their perception of interaction with the transcendent in daily life.

##### Inventory of Complicated Spiritual Grief

The Inventory of Complicated Spiritual Grief [89] measures how much individuals specifically consider the level of loss experienced when responding to indicators of spiritual crisis that affect their relationship both with God and with fellow worshipers. It is composed of 18 items (Annex 5) with a 5-point Likert response scale (0=not true at all and 4=absolutely true). The factorial analysis highlighted a 2-factor structure, as follows: (1) “Insecurity with God,” which is composed of 7 items that investigate the individual’s insecurity toward their relationship with God, and (2) “Disruption in Religious Practice,” which is composed of 11 items that investigate how far the individual has abandoned religious practices.

#### Qualitative Measurement (Interview)

##### Thematic Analysis

Thematic analysis [84] has been widely used in mixed methods design, because it can be applied to a broad range of epistemologies and research questions, enabling researchers who use different research methods to communicate with each other [90]. It is a method for identifying, analyzing, organizing, describing, and reporting themes identified within a qualitative data set [84], producing trustworthy and insightful findings [91].

### Sample Size Estimation

The primary endpoint of this study is the impact of the COVID-19 pandemic on the grief of mothers and fathers who experienced a perinatal loss during the pandemic. Considering a power (1-β) of 0.80 and a type I error (α) of .05, a sample of 34 parental couples is needed. The sample size is extremely low, but it will be in line with literature studies on perinatal loss that include and analyze small samples of couples [92,93]. As indicated by literature [94], the mixed methods design can help studies that involve small samples.

### Analysis

Quantitative and qualitative analysis will be carried out for the questionnaires and interviews. Appropriate data analysis will be performed using standard statistical packages.

#### Quantitative Data Analysis

The following steps will be performed: (1) descriptive analysis of all questionnaires prepared for the mother and father and evaluation of the differences between mother and father using 2-tailed *t* test for matched pairs. The results of the power analysis conducted using the GPower program indicate that comparison of the averages for fathers and mothers assuming an average effect, an alpha level of .05, and a power of .80 requires at least 34 couples; (2) evaluation of the change over time for mother and father between the first survey (after bereavement) and second survey (after 6 months) using repeated measured ANOVA. The results of the power analysis conducted using the GPower program indicate that comparison of the averages over time (2 measurements over time), for fathers and mothers assuming an average effect, an alpha level of .05, and a power of .80 requires at least 34 couples; (3) preliminary examination of the bivariate correlations between the measurements examined in the study and the sociodemographic variables using Pearson correlation; and (4) define a multiple regression model with the main predictive variables of the management of perinatal bereavement (including only the variables found to be significant in the preliminary examination). The results of the power analysis conducted using the GPower program indicate that a multiple regression model capable of explaining a significant share of the variability of the scores of the dependent variable, assuming a medium-sized effect, an alpha level of .05, and a power of .80, requires at least 92 people, including 5 predictors in the model, and at least 118 people, including 10 predictors in the model. Finally, if we manage to reach the number of 100 participants, estimating between 10% and 15% attrition, we will be able to proceed as described.

#### Qualitative Data Analysis

Participants (ie, the father and mother) will be asked to sit a semistructured interview that will further explore the issues investigated by the questionnaires. Parent interviews will be carried out separately via the Zoom platform. The interviews will be fully recorded and transcribed verbatim to be analyzed with the support of the Atlas.ti software. A thematic analysis will be carried out on the transcripts to identify the main common themes among the interviewees. We will focus on recognizable convergences and specificities through an appropriate comparison of the texts. The emerging themes identified within the experiences narrated by the participants will allow researchers to create a shared codebook within which the sentences stated by the participants will be assigned to a category according to the identified theme. The analysis will follow the 6 basic phases of preparation, generation of categories or themes, data encoding, verification of emerging understanding, search for alternative explanations, and drafting of the report. To verify the accuracy of the analysis and the interpretative procedures adopted by the interviewer and the supervisor, 2 other members of the research team will work on the texts until an agreement is reached between all researchers. The Atlas.ti software will be used to facilitate the identification of themes and will facilitate the creation of network graphics to describe the logical relationships between the concepts and categories identified by the researchers.

### Ethics Approval

Our study protocol was reviewed and approved by the Ethics Committee of Psychological Research, University of Padova (N. 3989 - 09/02/2021), and by the Institutional Ethics Board of the Spedali Civili of Brescia, Italy (N. NP4858 - 07/10/2021). All procedures performed in this study are in accordance with the ethical standards of the Institutional Ethics Board of the Spedali Civili of Brescia, and with the Declaration of Helsinki 1964 and subsequent amendments. We shall obtain written consent from the parents.

### Patient and Public Involvement

The parents were not involved in the design, conduct, reporting, or dissemination plans of this research.

### Confidentiality Procedure and Access to Data

Personal information about potential and enrolled participants will be collected only by members of the research team and cannot be accessed by other individuals. Personal information and survey data will be pseudonymized using an identification number. Only authorized study personnel will have access to any of the data associated with this study.

## Results

According to our sample size calculation, we expect that at least 34 couples from health care centers located in Italy will participate in the study.

We will publish all results in peer-reviewed international journals indexed in Web of Science or Scopus databases and present them at national and international conferences.

## Discussion

### Overview

The COVID-19 pandemic forced health services to redefine perinatal bereavement care protocols [78,79] due to the restrictions imposed to curb the spread of the virus. However, health care professionals were unprepared for these changes [31,32], which led to an increase in perinatal affective disorders in mothers and fathers [70], who felt isolated and lacking social support [36,37] at such a challenging time. To our knowledge, the impact of COVID-19 on care following the death of a baby has not been sufficiently explored.

This multicenter study will contribute to optimize the scientific knowledge in the field of studies of the impact of the COVID-19 pandemic on pregnancy and particularly on mothers and fathers grieving for a perinatal loss. It will contribute to the understanding of the psychological processes related to perinatal loss, bereavement care, and mourning during the COVID-19 pandemic; it will also provide information useful to preventing the risk of prolonged and complicated grief and parent psychopathologies and will promote perinatal mental health.

Regarding the implications in clinical practice, it would seem important to implement psychological services in health care centers (eg, counseling centers and obstetrics and gynecology wards) that can offer adequate support to mothers, fathers, and families who are experiencing the unexpected and painful event of perinatal loss of their child, especially if this happens in difficult and complex situations such as a global health emergency.

This study could pave the way for future scientific research in the same or similar area of interest that should consider perinatal bereavement, an event still poorly investigated and not always socially recognized, to develop a strong support system for the affected mothers, fathers, and families.

### Limitations

The most likely limitation of this study could be that some parents contacted by health care professionals may not agree to participate because the perinatal loss event may have been too traumatic and painful, and they may not want to talk about it anymore after it happened. Concerning data collection time point, recruited parental couples may not have experienced perinatal loss in the same week of pregnancy or in the same neonatal period, and this could be another limitation of this study. What the sample couples share is that the perinatal loss event occurred during the COVID-19 pandemic. Lastly, the sample, composed only of parents who speak and understand the Italian language, may be too small to be able to generalize the results.

## Data Availability

Data sharing does not apply to this article as data sets will not be generated or analyzed in this article.
